# Immune dysregulation and macrophage polarization in peri-implantitis

**DOI:** 10.3389/fbioe.2024.1291880

**Published:** 2024-01-29

**Authors:** Yue Li, Xue Li, Danni Guo, Lingwei Meng, Xianghui Feng, Yi Zhang, Shaoxia Pan

**Affiliations:** ^1^ Department of Prosthodontics, Peking University School and Hospital of Stomatology and National Center for Stomatology and National Clinical Research Center for Oral Diseases and National Engineering Research Center of Oral Biomaterials and Digital Medical Devices and Beijing Key Laboratory of Digital Stomatology and Research Center of Engineering and Technology for Computerized Dentistry Ministry of Health and NMPA Key Laboratory for Dental Materials, Beijing, China; ^2^ Stem Cell and Regenerative Medicine Lab, Beijing Institute of Radiation Medicine, Beijing, China; ^3^ Department of Periodontology, Peking University School and Hospital of Stomatology and National Center for Stomatology and National Clinical Research Center for Oral Diseases and National Engineering Research Center of Oral Biomaterials and Digital Medical Devices and Beijing Key Laboratory of Digital Stomatology and Research Center of Engineering and Technology for Computerized Dentistry Ministry of Health and NMPA Key Laboratory for Dental Materials, Beijing, China

**Keywords:** peri-implantitis, macrophages, polarization, immune dysregulation, periodontitis

## Abstract

The term “peri-implantitis” (peri-implantitis) refers to an inflammatory lesion of the mucosa surrounding an endosseous implant and a progressive loss of the peri-implant bone that supports the implant. Recently, it has been suggested that the increased sensitivity of implants to infection and the quick elimination of supporting tissue after infection may be caused by a dysregulated peri-implant mucosal immune response. Macrophages are polarized in response to environmental signals and play multiple roles in peri-implantitis. In peri-implantitis lesion samples, recent investigations have discovered a considerable increase in M1 type macrophages, with M1 type macrophages contributing to the pro-inflammatory response brought on by bacteria, whereas M2 type macrophages contribute to inflammation remission and tissue repair. In an effort to better understand the pathogenesis of peri-implantitis and suggest potential immunomodulatory treatments for peri-implantitis in the direction of macrophage polarization patterns, this review summarizes the research findings related to macrophage polarization in peri-implantitis and compares them with periodontitis.

## 1 Introduction

Since Brånemark first made dental implants available in the 1960s, they have been the norm for those with edentulism and missing teeth (Brånemark et al., 1969; Brånemark et al., 1977; Albrektsson, Brånemark, Hansson and Lindström, 1981). However, peri-implantitis (PI) is an increasingly serious biological complication of oral implantology. Its prevalence increases with the duration of the implant (French, Ofec and Levin, 2021; Obreja et al., 2021). The term “peri-implantitis” (PI) refers to an inflammatory lesion of the mucosa surrounding an endosseous implant and a progressive loss of the peri-implant bone that supports the implant (Renvert, Persson, Pirih and Camargo, 2018). According to reports, it affects between 5% and 37% of implants and between 11% and 53% of patients (Fransson, Lekholm, Jemt and Berglundh, 2005; Roos-Jansåker, Lindahl, Renvert and Renvert, 2006; Renvert, Roos-Jansåker, Lindahl, Renvert and Rutger Persson, 2007; Koldsland, Scheie and Aass, 2010; Rinke, Ohl, Ziebolz, Lange and Eickholz, 2011).

Although the clinical and radiological manifestations of PI and periodontitis share many features, there are key differences in their clinical progression, histological features, and microbial composition, suggesting different pathogenesis (Carcuac and Berglundh, 2014). By using 16S pyrosequencing, Kumar et al. discovered that the peri-implant microbiome differs greatly from the periodontal microbiome with regard to both health and illness. Peri-implantitis is a microbiological heterogeneous infection predominantly brought on by Gram-negative bacteria (i.e., the dominant species are not the same in each individual) and is not as complex as periodontitis (Kumar et al., 2012).

When PI samples were compared to periodontitis samples, the region of inflammatory infiltration was more than twice as large in the PI samples, and there were also considerably more macrophages and plasma cells in the PI samples overall (Carcuac and Berglundh, 2014). In both PI and periodontitis lesions, plasma cells and lymphocytes predominate. However, PMN and macrophages take more percentage in PI than in periodontitis (Esposito et al., 1997; Gualini and Berglundh, 2003; Berglundh, Gislason, Lekholm, Sennerby and Lindhe, 2004; Berglundh, Zitzmann and Donati, 2011; Carcuac and Berglundh, 2014). The periapical tissue goes through a “self-limiting” process when the ligature is removed in which the connective tissue capsule divides the ICT from the bone in periodontitis, whereas in the peri-implant tissue, the ICT extends to the bone crest (Berglundh, Zitzmann and Donati, 2011).

Implants dysregulate the immune response in the peri-implant mucosa (PIM), as shown by the development of a mouse model of dental implants and experimental PI (Pirih et al., 2015; Koutouzis, Eastman, Chukkapalli, Larjava and Kesavalu, 2017; Tzach-Nahman, Mizraji, Shapira, Nussbaum and Wilensky, 2017; Heyman et al., 2018; Heyman et al., 2022). This “dysregulated homeostasis” or inflammatory condition of the PIM may be the cause of the implant’s greater vulnerability to infection and the swift elimination of supporting tissue after infection (Carcuac and Berglundh, 2014).

**FIGURE 1:**
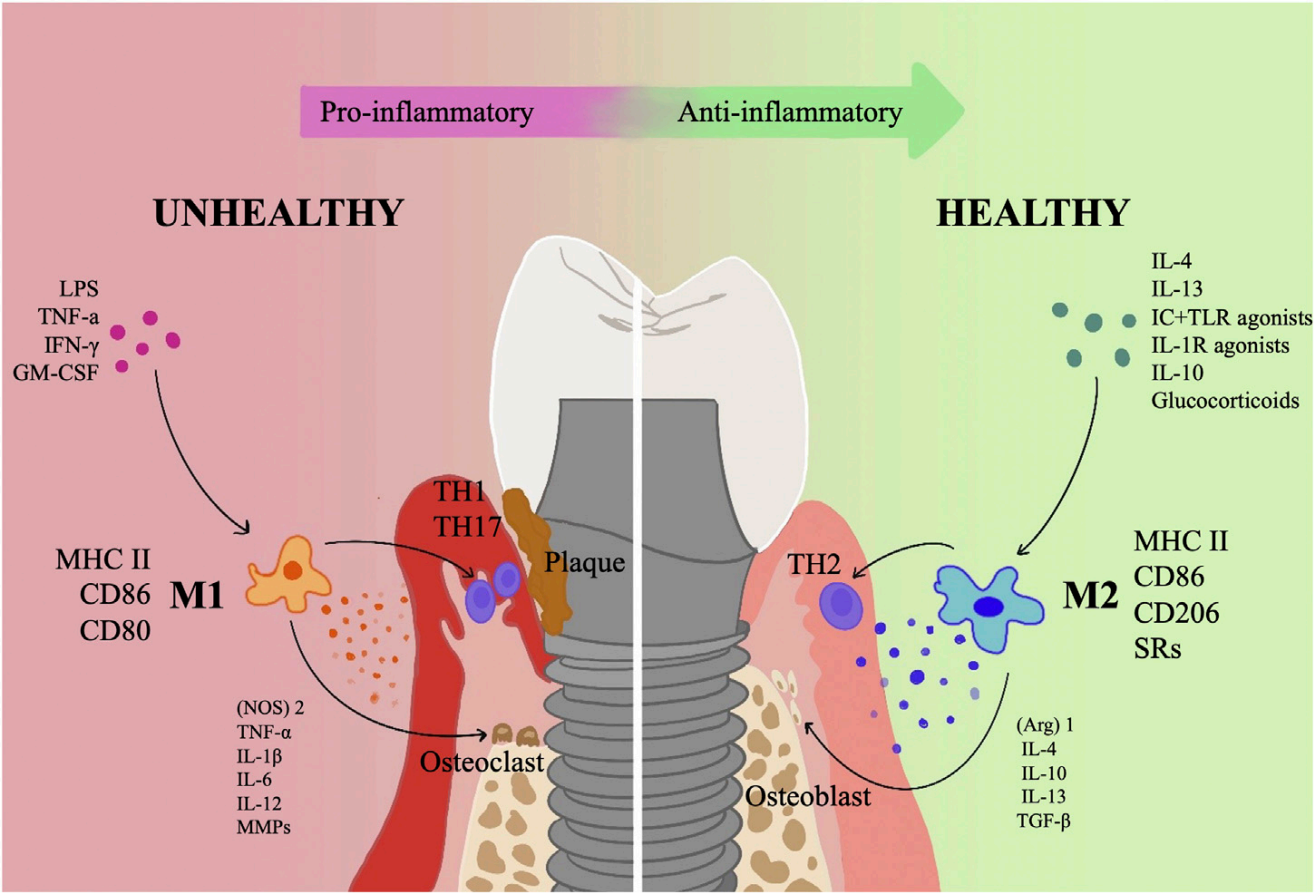
An overview of how polarized macrophages contribute to the incidence and growth of PI. The progressive and retreating phases of inflammation are dominated, respectively, by the M1 and M2 phenotypes of macrophages. M1 primarily serves a pro-inflammatory role, releasing a number of pro-inflammatory substances such (NOS)2, TNF-α, IL-1, IL-6, IL-12, and MMPs and collaborating with Th1 and Th17 cells. In addition, M1 type activates osteoclasts and causes resorption of alveolar bone; M2 type primarily functions as an anti-inflammatory, carrying out tissue repair via a variety of anti-inflammatory factors, such as (Arg)1, IL-4, IL-10, IL-13, and TGF-β, mainly synergizes with Th2 cells, and activates osteoblasts to promote bone regeneration.

Notably, Macrophages become polarized while responding to environmental signals, with M1 macrophages playing a role in bacterially-induced pro-inflammatory responses and M2 macrophages in inflammation regression and tissue repair (Yu et al., 2016; Palevski et al., 2017). Studies have shown an increase in polymorphonuclear leukocytes (PMN) and macrophages in PI lesions compared to periodontitis. Additionally, PI lesion samples revealed a notable rise in M1 macrophages (Fretwurst et al., 2020). This kind of macrophage polarization feature could partially explain the faster progression of PI in humans compared to periodontitis. It is consistent with the finding that PI advances more quickly than periodontitis because there is an increased quantity and density of PMN and macrophages (particularly M1) in the peri-implant lesions (Dionigi, Larsson, Carcuac and Berglundh, 2020). Studies on the function of macrophage polarization in the onset of PI and periodontitis have gradually risen in recent years (Figure 1). This review summarizes the research results related to macrophage polarization in PI and compares them with periodontitis in an attempt to deepen the understanding of the pathogenesis of PI and propose possible immunomodulatory therapies for PI in the direction of macrophage polarization patterns. This will improve our knowledge of, capacity to avoid, and manage PI.

## 2 Overview of the polarization of macrophages

Macrophages were first recognized for their phagocytic abilities. They also contributed to host-microbe equilibrium, antigen presentation, mobilization of immune defense mechanisms, and resistance to bacterial infection (Sun X. et al., 2021). Although several attempts have been made to classify macrophages, the most widely accepted classification has been the mononuclear phagocytic system (MPS). There are also other functional classifications of macrophages, for example, binary classification of inflammatory states classifies macrophages into activated macrophages and alternatively activated macrophages (AAM) (Gordon and Martinez, 2010; Sica and Mantovani, 2012; Wynn, Chawla and Pollard, 2013).

Macrophages can adjust to a variety of activation states that fall under the M1/M2 phenotypes of macrophage polarization in order to support immune activity and maintain tissue homeostasis (Martinez, Sica, Mantovani and Locati, 2008). The pro-inflammatory cytokines TNF-γ, interleukins IL-1, IL-6, and IL-12, as well as a high volume of reactive nitrogen and oxygen intermediates, are all produced by M1 macrophages after being primed by the interferon IFN-γ. These responses encourage Th1 responses with potent bactericidal and antitumor activity. IL-4 or IL-13 can prime M2 macrophages, which then express high levels of a metabolic marker called arginase (Arg) 1, the differentiation cluster CD206, and the anti-inflammatory cytokine IL-10, thereby dampening the inflammatory response to preserve tissue homeostasis, thereby attenuating the inflammatory response to maintain tissue homeostasis. The repression of parasites, stimulation of tissue remodeling, advancement of tumors, and immunomodulatory actions are all facilitated by M2 macrophages. Table 1 summaries the polarization types, characteristics and basic functions of macrophages. In summary, M1 macrophages have a role in bacterial killing and inflammation, whereas M2 macrophages are primarily involved in tissue homeostasis, suppression, inflammatory regression, and tissue healing (Morris, Singer and Lumeng, 2011; Sica and Mantovani, 2012).

**TABLE 1:** The polarization types, characteristics and basic functions of macrophages (Sima and Glogauer, 2013; Sun X. et al., 2021).

Phenotypes	Stimuli	Special surface receptor	Cytokines	Basic function
M1	LPS	MHC II	IL-1β	Stimulates the endothelium of vessels
	TNF-a	CD86		Makes lymphocytes active
	IFN-γ	CD80		Localized deterioration of tissues
	GM-CSF			Enhances effector cell accessibility
				Generation of IL-6
			IL-6	Activation of lymphocytes
				Increased synthesis of antibodies
				Stimulates the synthesis of acute-phase proteins
			TNF-α	Enhances the permeability of the vascular endothelium
				Enhanced fluid drainage to lymph nodes
				Enhanced entrance of IgG, complement, and cells into tissues
				Metabolite mobilization
			CXCL8/IL-8	Chemotactic factor that attracts T-cells, basophils, and polymorphonuclear neutrophils to the infection site
				Degranulates, mobilizes, and activates polymorphonuclear neutrophils
			IL-12	Triggers the activation of natural killer cells
				Stimulates the development of CD4^+^ T cells into T-helper 1 cells
			IL-23	Stimulates the generation of interferon gamma and T-helper 17 memory T-cell proliferation
			CCL2/monocyte chemotactic protein-1	Attracts T-cells, monocytes, basophils, immature dendritic cells and natural killer cells
			CXCL9	Involved in T-cell trafficking
			CXCL10	Attracts natural killer cells and T-cells
				Signals through CXCR3
M2	IL-4	MHC II	IL-1R antagonist	Acts as a natural antagonist of IL-1 function
	IL-13	CD86	IL-10	Inhibits the production of pro-inflammatory cytokines, including granulocyte–macrophage colony-stimulating factor, TNF-α, IFN-γ, IL-2, and IL-3
	IC + TLR/IL-1R agonists	CD206	TGF-β1	Inhibits cell growth
	IL-10 Glucocorticoids	SRs		Anti-inflammatory
				Induces switch to IgA production
			Insulin-like growth factor-1	Stimulates fibroblast proliferation and survival
			CCL17	Attracts T-cells and macrophages
			CCL18	Attracts lymphocytes, immature dendritic cells and monocytes
			CCL22	Attracts T-helper 2 cells and other CCR4-expressing cells
			CCL24	Attracts T-helper 2 cells

The development of numerous inflammatory disorders, including infections, obesity, and cancer, is characterized by an imbalanced M1/M2 ratio (Wynn et al., 2013). Obesity, atherosclerosis, diabetes, allergies and asthma, autoimmunity, and cancer are a few examples of chronic diseases that are linked to specific macrophage polarization profiles (Sima and Glogauer, 2013). It has been proven that macrophages can become functionally polarized *in vivo*, both in healthy and unhealthy circumstances. Pregnancy, embryogenesis, and the preservation of normal conditions in particular tissues (such as the testis and fat tissue) are all included in the former. Included in the latter are cancer, vascular disease, infection, chronic inflammation, tissue healing, and metabolism (Sica and Mantovani, 2012).

## 3 Polarization of macrophages in periodontitis

As the sixth most common disease in the world, periodontitis is a common condition that affects many people. In its extreme stages, 10% of adult population are affected (Larsson et al., 2022). It is a chronic infectious illness characterized by microbial-related and host-mediated inflammation, which is brought on by the persistent breakdown of supportive periodontal tissues, which is started by plaque biofilm (Tonetti, Greenwell and Kornman, 2018). A considerable number of animal experiments and human studies have shown increased polarization of M1 macrophages in periodontitis (Table 2). In mice infected with Porphyromonas gingivalis (Pg), an animal investigation found that M1 macrophages dramatically expanded in the gingival tissue (Lam et al., 2014). M1 macrophages increased higher than M2 in the periodontitis group compared to the healthy control group, according to research by Yu T et al. on animals. Additionally, periodontal tissue affected by periodontitis showed an increase in the M1 inflammatory factors TNF-α and IL-1β as well as the M2 inflammatory factor IL-10 (Yu et al., 2016). Another human study showed that M1 macrophages increased in periodontitis compared to healthy controls (Higuchi, Sm, Yamashita, Ozaki and Yoshimura, 2020). However, when periodontitis worsens, the macrophage phenotype may alter. M1 is enhanced during the inflammatory phase while macrophage phenotype polarizes towards the M2 type during the recovery stage (Gonzalez et al., 2015; Viniegra et al., 2018; Zhou et al., 2019; Wu X. et al., 2020).

**TABLE 2:** Studies related to macrophage polarization in periodontitis.

Author & year	Article type	Polarization markers		Possible regulatory pathways	Results
		M1	M2		
Lam et al. (2014)	Animal study (mice)	CD86	CD206	—	Pg infection causes functional/inflammatory M1 macrophage infiltration of gingival tissue and alveolar bone resorption. M1 macrophages (CD86^+^), but not M2 macrophages (CD206+), are the predominant macrophage phenotype in gingival infiltration
Gonzalez et al. (2015)	Animal study (Rhesus monkeys)	M1 gene profiles	M2 gene profiles	—	Age and periodontitis cause a large rise in macrophages. The M1 phenotype is the most common rise in older, particularly in tissues with periodontitis
Yu et al. (2016)	Animal study	(NOS)-2	CD206	In the setting of periodontitis, a multitude of signals, such as pro- and anti-inflammatory cytokines upregulated in the macrophages themselves, as well as M1-stimulating (IFN-γ) and M2-stimulating (IL-4) cytokines upregulated in T-helper cells, may combine to generate a macrophage phenotype	In the periodontal tissues, the periodontitis group had a 14-fold increase in M1 type, a 4-fold rise in M2 type, and an improved M1/M2 ratio (*p* < 0.01) in comparison to the control group. Increased M1 and M2 macrophage phenotypes were linked to periodontal inflammation; the transition from M2 to M1 may be a major mechanism generating periodontal tissue damage, including alveolar bone loss
Viniegra et al. (2018)	Animal study (mice)	TNF-α	IL-10	—	M2 activation, partly via direct action on osteoblasts, promotes bone repair during healing of periodontal lesions. In osteolytic illness, immunomodulation of macrophages to polarize them toward the M2 type stimulates bone growth
			TGF-β		
			CD206		
Zhou et al. (2019)	Human study	iNOS	CD206	—	The periodontitis group had considerably higher levels of TNF-α, IFN-γ, IL-6, and IL-12, along with a larger M1/M2 ratio and a greater number of M1 cells when compared to the control group
Wu et al. (2020b)	Human study	CD86	CD163	Akt2/JNK1/2/c-Jun Akt2/miR-155–5p/DET1/c-Jun	Inhibition of Akt2 promotes macrophage M2 polarization and rescues periodontitis-induced bone loss
Ahmad, Naqvi, Valverde and Naqvi (2023)	Animal (mice) and Human study	iNOS	ARG1	LncRNA MALAT1/microRNA-30b	MALAT1 functions and is expressed antagonistically with miR-30b, another non-coding RNA. MALAT1 knockdown favors the M2 phenotype, while miR-30b overexpression encourages M2 polarization
		STAT1	STAT3		
		TNF-α	CCL2		
		ARG2	IL-10		
(Wu, Wang, Chen, Wang and Gu, 2023)	Animal study (mice)	CD86	CD206	PTEN/Akt1/Akt2	M2 polarization is induced in macrophages by PTEN inhibition, while M1 polarization is promoted by PTEN overexpression. PTEN inhibitor therapy prevented alveolar bone resorption and markedly decreased the local inflammatory state in mice
(Yang et al., 2023)	Animal studu (mice)	iNOS	CD206	IL-37/NLRP3	In the gingival tissues of periodontitis-stricken mice, IL-37 markedly decreased the number of iNOS + cells while increasing the number of CD206+ cells.By preventing the activation of the NLRP3 inflammasome and facilitating the polarization of M1/M2 macrophages, IL-37 stopped the advancement of periodontitis
Li et al. (2023a)	Animal study (mice)	iNOS	Arg-1	MicoRNA-126/MEKK2	By controlling the MEKK2 signaling pathway, miR-126 inhibits macrophage M1 polarization and stops alveolar bone resorption in individuals with diabetic periodontitis

### 3.1 M1 macrophage polarization in periodontitis

Numerous M1 macrophages are present at the sites of bone degradation in chronic osteolytic disorders, such as various types of arthritis and periodontitis. These macrophages contribute significantly to disease-induced bone resorption by producing inflammatory cytokines including IL-1β and TNF-α and activating osteoclasts (Arend and Dayer, 1990; Stashenko, Jandinski, Fujiyoshi, Rynar and Socransky, 1991; Metzger, 2000; Górska et al., 2003; Andrukhov et al., 2011; Shaddox et al., 2011). Clinical outcomes may be enhanced by antagonist therapy that lowers TNF-α and IL-1β levels (Zwerina, Redlich, Schett and Smolen, 2005; McInnes and Schett, 2007). By employing IL-1β and TNF-α antagonists or knocking down the IL-1 receptor and TNF receptor, alveolar bone resorption in mice with experimental periodontitis was also decreased (Assuma, Oates, Cochran, Amar and Graves, 1998; Graves and Cochran, 2003). Additionally, gingival crevicular fluid IL-1 levels were found to be lower, IL-10 levels were higher, and bone resorption activity was lower when periodontal treatment was effective (Holmlund, Hänström and Lerner, 2004; de Lima Oliveira et al., 2012).

Matrix metalloproteinases (MMPs), which are involved in the breakdown of the extracellular matrix, are just one of the significant proteases that M1 macrophages release in addition to cytokines in the advancement of periodontal disorders (Franco, Patricia, Timo, Claudia and Marcela, 2017). MMPs are produced as a result of the inflammatory cytokines TNF-α, IL-1, and IL-6, all of which are highly expressed in diseased periodontal tissue (Stashenko et al., 1991; Irwin and Myrillas, 1998; Irwin, Myrillas, Traynor, Leadbetter and Cawston, 2002), some of these MMPs are also associated with increased M1/M2 ratios during disease (J. Yang et al., 2018).

### 3.2 M2 macrophage polarization in periodontitis

Widespread expression of the M2 macrophage’s IL-10 in inflamed periodontal tissue is linked to tissue healing, a reduction in periodontitis severity, and a reduction to inflammation (Lappin, MacLeod, Kerr, Mitchell and Kinane, 2001; Garlet, Martins, Fonseca, Ferreira and Silva, 2004; Garlet, 2010). In IL-10 deficient animals, which were more vulnerable to Pg-induced alveolar bone loss, its protective effect was also demonstrated (Sasaki et al., 2004). Additionally, TGF-β is regarded as one of the most significant cytokines involved in the upkeep of the M2 phenotype, which suppresses the synthesis of endogenous NO (Vodovotz, Bogdan, Paik, Xie and Nathan, 1993), and is crucial for the recruitment of bone marrow mesenchymal stem cells (MSCs) during tissue regeneration (Fu et al., 2019). By releasing IL-4, IL-10, IL-13, and TGF-β throughout the inflammatory process, M2 macrophages counteract the M1 type macrophage response, control inflammation, and aid in tissue repair and wound healing (Mosser and Edwards, 2008; Wynn and Vannella, 2016).

### 3.3 Potential pathways for macrophage polarization in the etiology of periodontitis

M1 macrophages and Th1/Th17 lymphocytes are more prevalent than M2 macrophages and Th2/Treg lymphocyte subsets in active periodontal diseases compared to both of these cell types (Cavalla and Hernandez, 2022).

Through interactions with other immune cells, it has been demonstrated that macrophage polarization plays a role in the etiology of periodontitis: (1) macrophage-PMN-monocyte crosstalk: during inflammation, M1 macrophages locally recruit PMN to clear pathogens. Monocytes emerge after PMN recruitment and are activated as M2 macrophages to remove apoptotic PMN and other debris; (2) macrophage-lymphocyte crosstalk: M1 type macrophages activated by LPS, TNF-α, and IFN-γ produce IL-23, which stimulates Th17 cell infiltration. An inflammatory amplification loop is created when a Th17 cell releases IL-17 (a pro-inflammatory cytokine that promotes PMN recruitment and activation), IL-1, IL-6, TNF-α, MMPs, and RANKL. The decoy receptor osteoprotegerin and RANKL, a significant pro-osteoclastic mediator, are necessary for the coupling of bone resorption and creation (Sima and Glogauer, 2013).

## 4 Relationship between implants and dysregulated immune responses in the peri-implant mucosa (PIM)

Animal experiments based on a murine implant model have shown that the titanium implant itself promoted peri-implant inflammation and dysregulated mucosal homeostasis. Langerhans cells, the primary antigen-presenting cells of the oral epithelium, were hampered in their ability to mature, which was a result of the implant’s release of titanium ions. Titanium dental implants disrupted the immunological control of the PIM by impairing the growth of oral Langerhans cells (Heyman et al., 2018).

In peri-implant tissue biopsies, a reduction in inflammatory cell density was seen as healing time increased, so it is thought that the onset and regression of inflammation is a characteristic of PIM healing (Tomasi et al., 2016). This occurrence might be the PIM’s transitional immunological state before it returns to a homeostatic level resembling healthy gingival tissue. However, inflammatory infiltration of the PIM had been reported in implants that did not show clinical signs related to inflammation even 6 months after implant insertion, as found in animal studies (Pongnarisorn et al., 2007). Determining whether the PIM reaches a “normal” steady state, as it does in the gingiva, is therefore uncertain, suggesting the possibility that the PIM develops an alternative immune homeostasis. Given that the peri-implant tissue is more “inflamed” than the normal gingiva based on Th17/Treg homeostasis, this theory could explain why the implant is more susceptible to infection (Heyman et al., 2022). As mentioned previously, M1 macrophages and Th1/Th17 lymphocyte subsets are more prevalent than M2 macrophages and Th2/Treg lymphocyte subsets in active periodontal diseases (Cavalla and Hernandez, 2022). Thus, although the role played by macrophage polarization in the immune dysregulation of peri-implant tissues has not been fully investigated, it can be hypothesized that its role should not be underestimated.

Additionally, utilizing a mouse dental implant model, Heyman et al. discovered that dental implants were able to promote dysbiosis of the oral microbiota and increase inflammation and bone loss in the remote teeth in addition to locally raising inflammation and bone loss. It was not entirely clear which mechanisms induced the promotion of bone loss at the remote site. The Th1 immune response, represented by IFN-γ, may yet be implicated in this process, according to findings of cytokine production and lymphocyte infiltration in the gingiva (Heyman et al., 2020). The possibility of M1 macrophages contributing is also raised by this.

## 5 Polarization of macrophages in peri-implantitis

Currently, there are only several studies investigating macrophage polarization in PI. No consensus has been reached.

It was reported earlier that the number of M1 macrophages present was similar between the periodontitis and PI groups, although higher than that of healthy controls (Karatas et al., 2020). M1 and M2 expression in PI samples did not show any statistically significant differences (Galarraga-Vinueza, Obreja, Khoury, et al., 2021a).

However, research from the previous 2 years revealed that PI had much more M1 macrophages than periodontitis did. In comparison to periodontitis samples, it was discovered that PI samples showed a much greater degree of inflammatory cell infiltration and a significantly higher number of M1 macrophages (Dionigi et al., 2020; Fretwurst et al., 2020). M2 macrophage counts, however, did not significantly differ between the two illnesses (Fretwurst et al., 2020). M1 macrophage levels were also noticeably greater in advanced PI cases (i.e., radiographic marginal bone loss >50% of implant length, PI severity classification (Monje et al., 2019)), and a significant association between higher M1 macrophage expression and deeper probing depth was found (Galarraga-Vinueza, Obreja, Ramanauskaite, et al., 2021b). Table 3 summarizes the literature related to macrophage polarization in human PI in recent years.

**TABLE 3:** Studies related to macrophage polarization in peri-implantitis.

Author & year	Sample size		Inclusion criteria of patients with PI	Polarization markers		Results
	Number of patients with periodontitis	Number of patients with PI		M1	M2	
Fretwurst et al. (2016)	—	12	Severe peri-implant disease with indication for explantation included radiographic bone loss of more than two-third of the implant length, suppuration, mobility, or cortical bone perforations	PGM-1^1^	—	M1 macrophages were few overall in the specimens, and immunohistological analyses revealed that they concentrated in regions with higher amounts of the metals titanium and iron
Karatas et al. (2020)	15	15	2017 World Workshop (Berglundh et al., 2018)	iNOS	—	In comparison to periodontitis and PI specimens, peri-implant mucositis showed reduced iNOS expression, with no differences found in the former two
Fretwurst et al. (2020)	7	7	2017 World Workshop (Berglundh et al., 2018)	iNOS	CD206	M1 macrophage population was significantly increased in PI samples compared to periodontal disease samples (*p* < 0.01); M2 macrophage polarization showed similar levels in both (*p* > 0.05). In comparison to periodontitis specimens, the area and density of iNOS-positive cells in PI specimens were higher
Dionigi et al. (2020)	40	40	severe peri-implantitis:The subjects in this group demonstrated ≥1 implant with peri-implant bone loss ≥3 mm and a peri-implant probing pocket depth ≥7 mm, with bleeding on probing and/or suppuration (Carcuac and Berglundh, 2014)	iNOS	—	The area and density of iNOS-positive cells in PI specimens were greater than in periodontitis specimens
Galarraga-Vinueza, Obreja, Ramanauskaite, et al. (2021a)	—	20	the presence of at least one screw-type (one- or two-part) titanium implant diagnosed with peri-implantitis and indicated for surgical peri-implantitis treatment	CD80	CD206	M1>M2 (*p* = 0.01)
				CD68	CD68	
Galarraga-Vinueza, Obreja, Khoury, et al. (2021b)	—	14	2017 World Workshop (Berglundh et al., 2018)	CD80	CD206	M1>M2 (*p* = 0.16)

^a^ Legend: PGM-1: Glucose phosphate metastase-1. World Workshop (Berglundh et al., 2018): (1) Presence of bleeding and/or suppuration on gentle probing. (2) Increased probing depth compared to previous examinations. (3) Presence of bone loss beyond crestal bone level changes resulting from initial bone remodeling. Epidemiological studies need to take into account the error of measurements in relation to assessments of bone level changes. Bone loss should be reported using thresholds exceeding the measurement error (mean 0.5 mm).

### 5.1 M1/M2 polarization in regulating osteoclast and osteoblast functions

It is now believed that the large number of macrophages and elevated M1 macrophages observed in PI lesions indicate a strong immune system response to local factors that increase tissue destruction. The histological data in the literature are consistent with the progression of PI disease observed in the clinic (Derks et al., 2016; Fretwurst et al., 2020). The higher expression of M1 macrophages may be associated with a “destructive” inflammatory response and significant peri-implant osteolysis in advanced PI cases (Garlet and Giannobile, 2018; Zhuang et al., 2019; Fretwurst et al., 2020).

Through the secretion of cytokines that activate osteoclast precursors and encourage Th1 responses, M1 contributes to the activation of osteoclasts. Concurrently, M1 contributes to the generation of cytokines that are thought to be important for bone resorption, including PGE2, IL-1β, TNF-α, IL-6, and IL-12. PGE2 is the most potent inducer of periodontal bone resorption among them. It also facilitates a number of detrimental processes in the alveolar bone, including reducing osteoblast viability and mineralization and promoting the development of osteoclasts (Oka et al., 2007; Ruiz-Heiland, Yong, von Bremen and Ruf, 2021). LPS stimulates M1’s expression of IL-1β, and TNF-α and IL-1β together stimulate M1’s synthesis of IL-1β to support osteoclast activation and differentiation (Ruiz-Heiland et al., 2021); TNF-α also causes T cells and B cells to produce RANKL (Becerra-Ruiz, Guerrero-Velázquez, Martínez-Esquivias, Martínez-Pérez and Guzmán-Flores, 2022). Furthermore, IL-6 causes osteoclasts to break down the extracellular matrix and create MMPs, which eventually results in alveolar bone resorption (Figueiredo et al., 2020).

As was previously noted, TGF-β is regarded as one of the key cytokines in the preservation of the M2 phenotype and is crucial for bone marrow MSC recruitment during tissue healing (Fu et al., 2019). M2 also expresses high levels of IL-10 (Zhou et al., 2019), which helps to partially explain its role in the formation of new bone. The excessive effects of IL-10 and IL-4 on the healing process appear to be related to the downregulation of proinflammatory cytokines and MMP as well as the stimulation of osteoblasts. M2 secretes BMP-2, which speeds up osteogenesis (Liang, Wang, Wu and Wang, 2021). To sum up, M2 secretes anti-inflammatory and repair mediators, including TGF-β, IL-4, IL-10, and vascular endothelial growth factor, which in turn suppress proinflammatory cytokines and encourage tissue regeneration and homeostasis restoration. The M2-induced local microenvironment promotes osseointegration and angiogenesis (Park, Silvin, Ginhoux and Merad, 2022).

### 5.2 Titanium particles and foreign body reactions in peri-implant tissues

The presence of foreign bodies is thought to be strongly associated with PI, and they cause a dysregulated immune response in the peri-implant tissues. These foreign bodies are mainly titanium and dental adhesives (Wilson et al., 2015). Successive studies have reported cases of post-implant titanium allergy or peri-implant mucosal reactive lesions, and metallic-like particles and cells suggestive of allergic reactions, such as eosinophils and PMN, have been observed histologically. Available data suggested that titanium particles were present in more than 90% of PI lesions (Shafizadeh, Amid, Mahmoum and Kadkhodazadeh, 2021). Several *in vitro* studies have confirmed that microns or nanoparticles of titanium implant alloys may be cytotoxic and enhance pro-inflammatory responses (Okuda-Shimazaki, Takaku, Kanehira, Sonezaki and Taniguchi, 2010; Cai et al., 2011; Irshad et al., 2013; Pettersson et al., 2017). A significant inflammatory reaction was seen in soft tissue biopsies near implants when titanium particles were present (Schlegel, Eppeneder and Wiltfang, 2002; Olmedo et al., 2012; Wilson et al., 2015). There was considerable evidence that debris, titanium ions, and particle shedding could lead to sterile peri-implant inflammation and implant failure (Revell, 2008).

Histological biopsies of human PI samples revealed that M1 macrophages accumulated in areas of increased titanium and iron concentrations (Fretwurst et al., 2016). It has been discovered that titanium particles cause macrophages to react similarly to LPS, and the resulting inflammatory response fuels osteoclast-mediated bone tissue destruction. *In vitro* and *in vivo* gene expression, secretome profiling, fluorescence activated cell sorting (FACS), and other analyses on macrophages revealed that M1 polarization occurs in response to titanium particles. However, all of their assays were performed during the early inflammatory phase. Inflammation regression was observed in some tissues *in vivo* after 6–8 weeks, indicating that M1 and M2 macrophages may be distributed more dynamically and intricately over time (Eger et al., 2018).

To study the impact of various titanium particle sources on macrophage polarization, Eger et al. used a mouse calvarial model (Eger, Sterer, Liron, Kohavi and Gabet, 2017). The findings demonstrated that there was no noticeable difference in M2 macrophage numbers between the experimental and control groups. However, mice exposed to titanium particles produced by machined (M) or sandblasted and acid-etched (SLA) processes had considerably more M1 macrophages (Eger et al., 2018).

*In vitro* macrophage cultures revealed similar results. TNF-α, IL-1β, and IL-6 mRNA expression in macrophages increased (up to a 3.5-fold rise) when TiO2 particles were added to the culture medium (Ramenzoni, Fluckiger, Attin and Schmidlin, 2021). Titanium ions in physiological solutions induced the release of IL-1β via activating inflammatory vesicles in human macrophages (Pettersson et al., 2017), and all these cytokine profiles were characteristic of M1 polarization.

In conclusion, immune dysregulation can be found in PI. The most common phenomenon is the polarization of macrophages, but related studies are still lacking. The difficulty of creating an animal model is a significant factor in the paucity of data regarding the etiology of PI. PI lesion tissue is not easily available may also be responsible for it. In addition, Regarding the indicators of M1/M2 polarization, there is currently no definite agreement in the macrophage polarization literature (Fretwurst et al., 2020). Research is still needed in the area of choosing more precise molecular markers to distinguish M1/M2 macrophages (Galarraga-Vinueza, Obreja, Ramanauskaite, et al., 2021a).

## 6 Immunoregulatory therapy for peri-implantitis linked to polarized macrophages

One should not undervalue the role that macrophage polarization plays in the clinical management of periodontitis and PI. It is currently thought that the major goal of macrophage polarization therapy is to get macrophages to polarize toward the M2 macrophages in order to reduce inflammation, encourage tissue repair, and produce anti-inflammatory benefits (Sun et al., 2021; Whitaker, Hernaez-Estrada, Hernandez, Santos-Vizcaino and Spiller, 2021). Promoting macrophage polarization from M1-type to M2-type by immunomodulatory therapy to promote bone regeneration has been successfully attempted in diabetic fracture healing models, and in bone-related diseases including osteoarthritis (OA), osteoporosis (OP), and bone defects (Whitaker et al., 2021; Wang et al., 2023). Macrophage polarization immunomodulatory therapy for periodontitis is currently a hot topic, but those therapies regarding PI are currently rare.

The current literature on regulating macrophage polarization as a therapeutic target for periodontal disease can be summarized as follows: (1) anti-cytokine therapy: when anti-TNF-α therapy was used in combination with mechanical debridement, periodontal parameters showed a tendency of improvement (Pers, Saraux, Pierre and Youinou, 2008; Mayer, Balbir-Gurman and Machtei, 2009; Ortiz et al., 2009). Therapeutic blocking of IL-1 receptors dramatically reduced local inflammatory cell infiltration, osteoclast activation, and bone resorption in an animal model of periodontitis (Assuma et al., 1998; Delima et al., 2001); (2) pharmacological treatment: when used systemically, rosiglitazone inhibited bone resorption during inflammation, increased bone regeneration during the repair of periodontitis, and polarized macrophages toward the M2 macrophages (Di Paola et al., 2006; Hassumi et al., 2009; Viniegra et al., 2018). Other drugs that affect macrophage polarization include PPARγ agonists (thiazolidinediones) (Charo, 2007; Stienstra et al., 2008; Lu et al., 2011), zoledronic acid, statins (Fujita et al., 2010), trabectedin (Germano et al., 2010); (3) cell therapy: isolated polarized M2 macrophages had the potential to initiate the regression of periodontal disease inflammation (Sima and Glogauer, 2013); (4) gene knockout: It has been shown that local injection of AKT inhibitors decreased the M1/M2 ratio and reduced alveolar bone resorption in mice with periodontitis, and that *in vitro* knockdown of Akt2 hindered M1 polarization and enhanced M2 polarization (Zhuang et al., 2019; Wu et al., 2020). The polarization of M1 macrophages was also decreased by TET1 knockdown because it prevented the NF-κB signaling pathway from being activated (Huang, Tian, Li and Xu, 2019).

**FIGURE 2:**
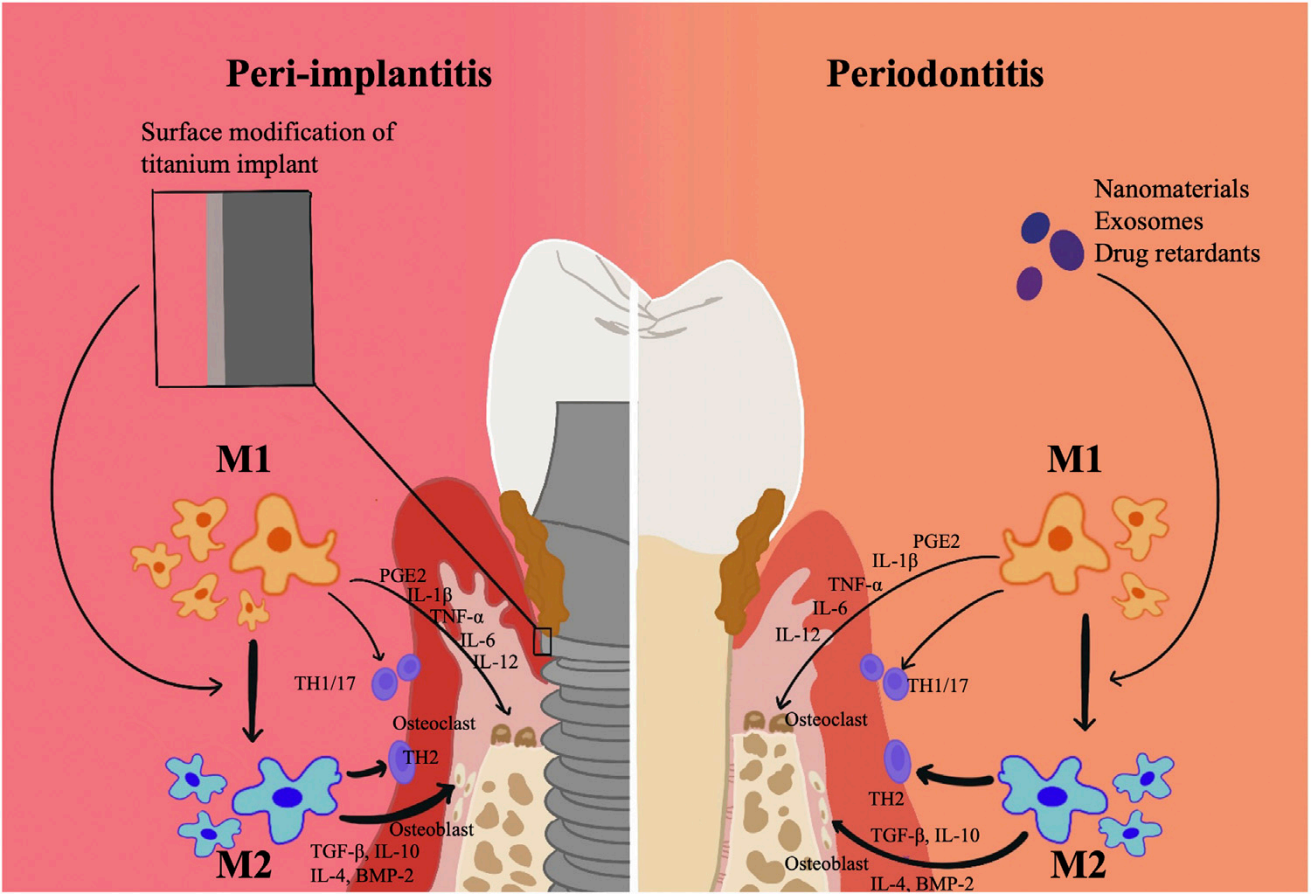
Surface modification technique of titanium related to Macrophage polarization in periodontitis and peri-implantitis. Surface modification of titanium may influence macrophage polarization. By modifying macrophage polarization, surface modification technique might reduce chronic inflammation and further increase osseointegration around the implant material.

Surface modification of titanium may influence macrophage polarization (Figure 2). Successfully synthesising IL-23R non-competitive antagonist nanocoatings on titanium surfaces, Pizarek et al. discovered that the coatings inhibited the IL-23/17A pathway, which is a source of inflammation, and polarized macrophages to the M2 phenotype in *vitro* cellular studies (Pizarek, Fischer and Aparicio, 2023). In a recent study, it was discovered that by modifying macrophage polarization, a surface modification technique using peptide coatings might reduce chronic inflammation and further increase osseointegration around the implant material (Bai et al., 2020). By interfering with integrin-α2β1 and integrin-αvβ3, peptide-modified titanium implants might successfully reduce peri-implant inflammation in wear particle models and induce macrophage polarization to a pro-healing M2 phenotype. With the use of tetravalent 3,4-dihydroxy-L-phenylalanine (DOPA) and Arg-Gly-Asp (RGD) sequences, this catecholic peptide with mussel-inspired structure was created. The mussel adhesion mechanism allowed for the easy apposition of this peptide to the surface of medical titanium materials, enhancing osteoblast adherence and fostering osteogenesis of titanium implants even under inflammatory circumstances (Guo et al., 2022).

In addition, MSC therapy has great potential in preventing and treating peri-implantitis. Li et al. used a hydrogel loaded with gingival-derived MSCs and injected it into the peri-implant area of a rat model of early implant placement and found that it was effective in improving epithelial closure around the implant and promoting M2 macrophage polarization. This would aid in preventing the growth of peri-implantitis (Li et al., 2023). However, the precise molecular processes and signaling pathways of interactions between epithelial cells and macrophages have not yet been clarified.

Nevertheless, the majority of inflammatory disease treatments, including those for periodontitis, are palliative and only offer temporary relief. The idea of immunomodulatory nanosystems (IMNs) may be able to solve this issue (Ahamad et al., 2021). The main IMNs for macrophage polarization-associated periodontitis include nanomaterials, exosomes, and periodontal drug retardants (Sun et al., 2021). Table 4 summarizes the immunomodulatory treatment strategies for macrophage polarization-associated periodontitis in recent years. Possible therapeutic strategies related to macrophage polarization in PI are less studied, but the current research in the periodontal field may provide some new directions for future research.

**TABLE 4:** Materials used in Immunomodulatory therapies related to Macrophage polarization in periodontitis.

Methods	Author & year	Materials
Nanomaterials	Ni et al. (2019)	45 nm gold nanoparticles (AuNPs)
	Garapaty and Champion (2019)	ligand presentation on rods
	Wu et al. (2020a)	modified zirconia surface
	Sun et al. (2021b)	cerium@Ce6 multifunctional nanocomposite
	Wang et al. (2021)	antioxidant drug quercetin onto nano-octahedral ceria
	Yang et al. (2021)	micro/nanomesh
	Ming et al. (2023)	sericin-hydroxyapatite nanoparticles (Se-nHA NPs)
	Xiao et al. (2023)	liposome-encapsulated indocyanine green (ICG) and rapamycin drug-delivery nanoparticle (ICG-rapamycin)
	Wang et al. (2023a)	AuAg-procyanidins (AuAg-PC)
	Huangfu et al. (2023)	resveratrol (RES)-20(S)-protopanaxadiol (PPD) (RES@PPD NPs)
	Chato-Astrain et al. (2023)	dexamethasone-loaded titanium micro particles (TiP) (Dex-TiP)
Exosomes	Wang et al. (2019)	exosomes secretion of periodontal ligament cells (PDLs)
	Curtale, Rubino and Locati (2019)	MicroRNAs: Mir-146a, Mir-125a and Mir-145–5p
	Shen et al. (2020)	dPSC-ExO-chitosan hydrogel (dPSC-ExO/CS)
	Nakao et al. (2021)	exosomes secretion of gingival tissue-derived MSCs
	He, Zhang and Lin (2021)	microrNA-125A-5P
	Luo et al. (2023)	CXCR4-miR126-Exo
	Deng et al. (2023)	Bio-GelMA@Bio-EX hydrogels-Exo
Drug retardants	Zhuang et al. (2019)	controlled-release microparticles (MPS)

## 7 Conclusion

Animal experiments based on a murine implant model have shown that the titanium implant itself promoted peri-implant inflammation and dysregulated mucosal homeostasis. Titanium ions that were released from the implant acted as a mediator in this process. It is currently thought that the onset and resolution of inflammation is a characteristic of PIM healing, but it is unclear whether the PIM achieves a “normal” stable state as in the gingiva, suggesting the possibility that the PIM develops alternative immune homeostasis. The available data indicate that macrophage polarization plays a significant role in the dysregulation of peri-implant immunity, despite the fact that the mechanisms behind this dysregulation are not fully understood.

Macrophage polarization has a complex and extensive variety of roles, with M2 macrophages primarily involved in tissue homeostasis, suppression and regression of inflammation, and tissue repair, and M1 macrophages promoting bacterial death and increasing inflammation. Although studies related to macrophage polarization in PI are not sufficiently thorough, the available literature suggests that the higher expression of M1 macrophages in PI compared to periodontitis may be associated with a “destructive” inflammatory response and significant peri-implant osteolysis in patients diagnosed with advanced PI. Furthermore, macrophage polarization toward the M1 phenotype may be caused by micron- or nano-sized particles of typical titanium implant alloys.

The ability to control immune homeostasis has been tentatively shown in some studies to be a promising therapeutic strategy. This is accomplished by carefully examining the mechanisms of action of various cytokines and mediators that regulate macrophage polarization and by controlling the ratio of macrophages with different polarization phenotypes to achieve a good balance between immune defense and tissue homeostasis.

However, the need for an experimental model and an unambiguous agreement on the markers to distinguish M1 from M2 polarization, which permits careful examination of this crucial issue, still exists. For further research, more PI lesion tissue needs to be gathered. The treatment options for PI macrophage polarization are few and will likely require more research in the future.
